# Physicochemical Characterization and Cytotoxic Activity Evaluation of Hydroxymethylferrocene:β-Cyclodextrin Inclusion Complex

**DOI:** 10.3390/molecules17056056

**Published:** 2012-05-21

**Authors:** Rosa Iacovino, Jolanda Valentina Caso, Filomena Rapuano, Agostino Russo, Marina Isidori, Margherita Lavorgna, Gaetano Malgieri, Carla Isernia

**Affiliations:** 1 Department of Environmental Science, Second University of Naples, via A.Vivaldi 43, 81100 Caserta, Italy; E-Mails: valentina.caso@unina2.it (J.V.C.); agostino.russo@unina2.it (A.R.); gaetano.malgieri@unina2.it (G.M.); carla.isernia@unina2.it (C.I.); 2 Department of Biological and Environmental Science, University of Sannio, via Port’Arsa, 11, 82100 Benevento, Italy; E-Mail: filomena.rapuano@unisannio.it; 3 Department of Life Sciences, Second University of Naples, via A.Vivaldi 43, 81100 Caserta, Italy; E-Mails: marina.isidori@unina2.it (M.I.); margherita.lavorgna@unina2.it (M.L.)

**Keywords:** β-cyclodextrin, hydroxymethylferrocene, inclusion complex, stability constant, phase solubility diagram, cytotoxicity

## Abstract

An inclusion complex of hydroxymethylferrocene (FeMeOH) with β-cyclodextrin (β-CD) was prepared in the solid state by different techniques such as physical mixture, coprecipitation, kneading and freeze-drying. The formation of the inclusion complex was confirmed by X-ray Powder Diffractometry and Fourier Transform-Infrared spectroscopy. In aqueous solution, the 1:1 stoichiometry was established by a Job plot. The inclusion complex formation was also investigated by NMR and the stability constant (K_b_) of the complex was determined to be 478 M^−1^, which is in agreement with that obtained with UV-Vis tritation (K_b_ = 541.3 M^−1^). The phase solubility study showed a diagram classified as B_S_ type and that the solubility of FeMeOH was slightly increased in the presence of β-CD. Furthermore, utilizing phase solubility diagram data, the K_b_ was estimated to be equal to 528.0 M^−1^. The cytotoxic activity of FeMeOH and its complexation product with β-CD was determined using the MTT-assay on MDA-MB-231 cell line, showing that the inclusion complex has a higher capability of inhibiting cell growth compared to that of pure FeMeOH.

## 1. Introduction

Cyclodextrins (CDs) are cyclic oligosaccharides produced by enzymatic action on starch. The most commonly used CDs contain 6, 7, or 8 units of glucose connected with α-1,4 bonds and are named α-, β- and γ-cyclodextrin, respectively [1,2]. It is well known that CDs have a toroidal shape with a hydrophobic surface inside and free hydroxyl groups on the two rims which render them capable of forming inclusion complexes with hydrophobic compounds of appropriate size and polarity in aqueous environments. The driving forces for the inclusion complexation of cyclodextrin (CD) with guest molecules are mainly hydrophobic interactions, electronic effects, van der Waals forces and steric factors. In addition, hydrogen-bond formation at the rim of the cavity may play an important role [3,4]. During the past two decades, CDs and their derivatives have attracted considerable interest in the pharmaceutical field because of their potential capability to form complexes with a variety of drug molecules. Many advantages of these complexes have been reported in the scientific literature: increased solubility, enhanced bioavailability, improved stability, masking of bad taste or odor, reduced volatility, transformation of liquid or gas into solid forms reducing side effects and formation of drug release systems [5,6,7,8]. Molecular encapsulation may occur both in the solid state and in solution. In the solid state, guest molecules can be enclosed within the cavity or may be aggregated to the outside of the cyclodextrin molecule. In solution state, we deal with an equilibrium between complexed and noncomplexed guest molecules. When a guest molecule gets incorporated within the cyclodextrin cavity changes in its physicochemical properties occur and the changes can be followed by different techniques [9,10,11,12,13]. 

Ferrocene and its various derivatives have been applied in many fields such as bioorganometallic and medicinal chemistry [14,15,16], synthetic organic chemistry, materials science and catalysis [17,18], due to their unique physical and chemical behaviors [19]. Several types of ferrocenyl compounds have been synthesized and evaluated in terms of anticancer properties [20,21,22,23]. The cytotoxicity of ferrocene-containing alcohols were determined by Shago and co-workers. They also demonstrated a definite structure-activity relationship between the number of methylene spacers that separate the ferrocenyl from the alcoholic group, and the cytotoxic activity [24].

**Figure 1:**
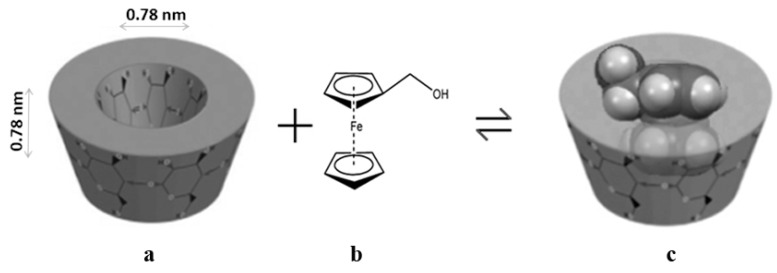
(**a**) Approximate dimensions of β-CD (**b**) molecular structure of FeMeOH and (**c**) schematic representation of FeMeOH:β-CD.

Hydroxymethylferrocene (FeMeOH), shown in Figure 1b, is probably the most studied and reacted compound in the monosubstituted ferrocene-containing alcohols class because of its ready availability [25]. In order to increase the solubility of FeMeOH in aqueous media we have employed β-CD to form noncovalent complexes. In the present work, we report the physico-chemical characterization of inclusion complex (FeMeOH:β-CD) between FeMeOH and β-CD (Figure 1). FeMeOH:β-CD solid systems in equimolar ratio were preparated by kneading, coprecipitation and freeze-drying methods. All the adducts were characterized by X-ray Powder Diffractometry (XRD) and Fourier Transform-Infrared spectroscopy (FT-IR). The stoichiometry and the stability constant of the complex were determined in solution utilizing the phase solubility diagram (PSD), Nuclear Magnetic Resonance (NMR) and Ultraviolet-Visible (UV-Vis) spectroscopy. Finally the cytotoxicity of FeMeOH and its complexation product were investigated using the MTT-assay on MDA-MB-231 breast cancer cells, an estrogen-independent cell line.

## 2. Results and Discussion

A guest molecule changes its physicochemical properties when it is incorporated within the cyclodextrin cavity. These changes provide methods to characterize whether guest molecules are really included or not in the cyclodextrin cavity [9]. In this work, the techniques used for the characterization of inclusion complexes between the guest and CDs can be divided in two groups:

1. solid state: XRD and FT-IR;
2. solution: Solubility studies, NMR, UV-Vis spectroscopy.

For ease in discussion, the samples are designated with different abbreviations: PM for physical mixture product, KND for the kneading product, CP for the coprecipitation product and FDY for the Freeze-drying product.

### 2.1. X-ray Powder Diffractometry (XRD)

**Figure 2:**
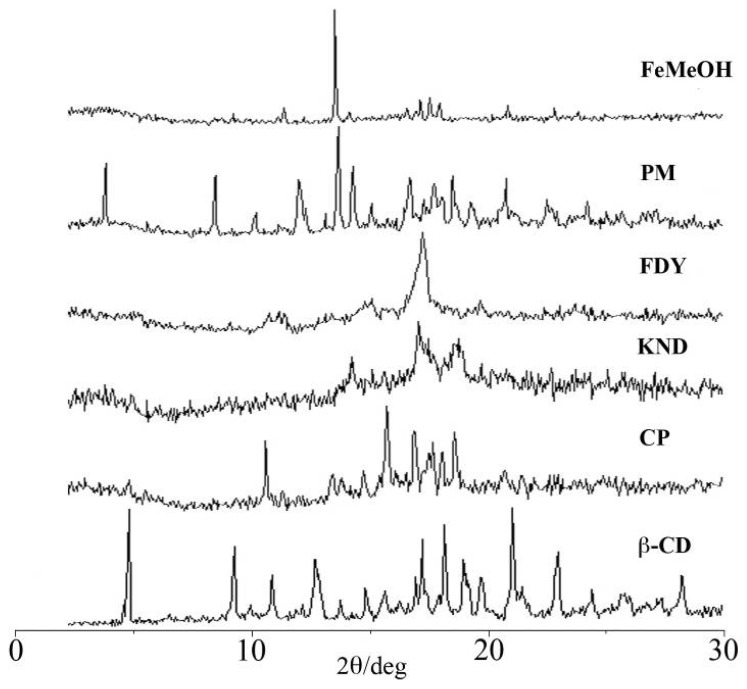
The powder XRD patterns of β-CD, CP, KND, FDY, PM and FeMeOH.

XRD diffraction studies are usually used to compare the crystallinity and amorphicity of compounds. Here they do not provide direct evidence of the formation of inclusion complex, but they are useful to detect any compound crystallinity changes upon host-guest interaction. XRD patterns of pure FeMeOH, β-CD, and of the PM, CP, KND, FDY samples are shown in Figure 2. The XRD pattern of β-CD revealed several diffraction peaks, which are indicative of its crystalline character. The five characteristic peaks of FeMeOH appeared at a diffraction angle of 2θ, at 11.99°, 14.12°, 17.64°, 17.97°, 18.41°, comparable to those reported in literature [26]. The appearance of new peaks at different 2θ, as well as the absence of the abovementioned characteristic peaks indicate the formation of a new crystalline phase upon complexation of FeMeOH with β-CD. The XRD pattern of CP did not contain peaks corresponding to β-CD or FeMeOH, and in fact it shows several new peaks which indicate the formation of a new crystalline phase as an indication of the inclusion complex formation [27]. For the KND product, a completely diffuse diffraction pattern was observed, which reveals its amorphous character. The XRD patterns of the FDY is characterized only by large diffraction peaks, in which it is no longer possible to distinguish the characteristic crystallinity peaks of pure FeMeOH or β-CD. These results indicate that FeMeOH is no longer present as a crystalline compound, and its β-CD solid complex exists in the amorphous state [28,29]. Comparing the diffraction patterns of pure components with PM, it is possible to observe that the PM diffractogram results in the combination of the components analyzed separately, with a marked diffraction peaks intensity decrease. 

### 2.2. Fourier Transform Infrared (FT-IR) Spectroscopy

**Figure 3:**
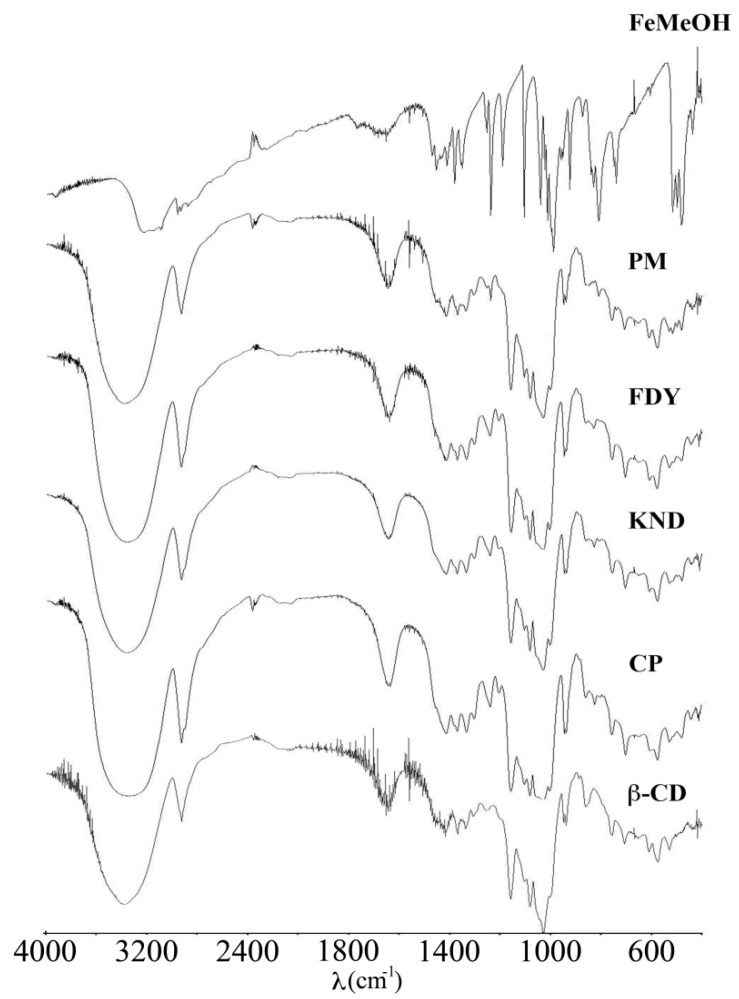
FT-IR spectra of β-CD, CP, KND, FDY, PM and FeMeOH.

FT-IR spectroscopy can be used to estimate the interaction, in the solid state, between CD and the guest molecules when the guests have some characteristic bands, such as hydroxyl, carbonyl or sulfonyl groups. The spectra of β-CD, FeMeOH, PM and FeMeOH:β-CD obtained by the three different techniques are shown in Figure 3. The spectra of the CP, KND, FDY and the PM samples are dominated by the vibrational bands of the β-CD since there are seven repeating glucose units in the molecule. The characteristic FT-IR absorption bands for β-CD are: 3,370 cm^−1^ (O-H stretching vibration); 2,925 cm^−1^ (O-H stretching); 1,648 cm^−1^ (O-H bending); 1,419 cm^−1^ (O-H deformation); 1,157 cm^−1^ (C-O-C stretching and O-H bonding); 1,029 cm^−1^ (C-O-C stretching). 

The FT-IR spectra of FeMeOH is consistent with the mono-substituted ferrocene structure, showing absorption bands at 1,012 and 1,104 cm^−1^ which are indicative that one of the two cyclopentadienyl rings is substituted [30]. FeMeOH has two pairs of bands in-plane С-Н vibrations at 1,012 and 988 cm^−1^ and out-of-plane С-Н vibration at 828 and 808 cm^−1^ [31]. Furthermore, the stretching band at 1,451 cm^−1^ is assigned to aromatic ring. In the FT-IR spectra of the CP, KND and FDY, the C-H stretching region of FeMeOH (3,226 cm^−1^) is covered by the O-H stretching band (3,370 cm^−1^) of β-CD. This last band results at 3,350 cm^−1^, showing a shift of 20 cm^−1^, in all compounds, due to hydrogen bonding with FeMeOH. The stretching of the C=C bond in the aromatic ring of FeMeOH (1,451 cm^−1^) disappears in the FT-IR spectra of the CP, KND and FDY, suggesting that the cyclopentadienyl ring enters into the cavity of β-CD.

### 2.3. Ultraviolet-Visible (UV-Vis) Spectroscopy

UV-Vis spectroscopy detects the complexation by a change in the absorption spectrum of the guest molecule. The modification in peak intensity are assumed to result from changes in the solvent microenvironment upon inclusion of the guest [32].

**Figure 4:**
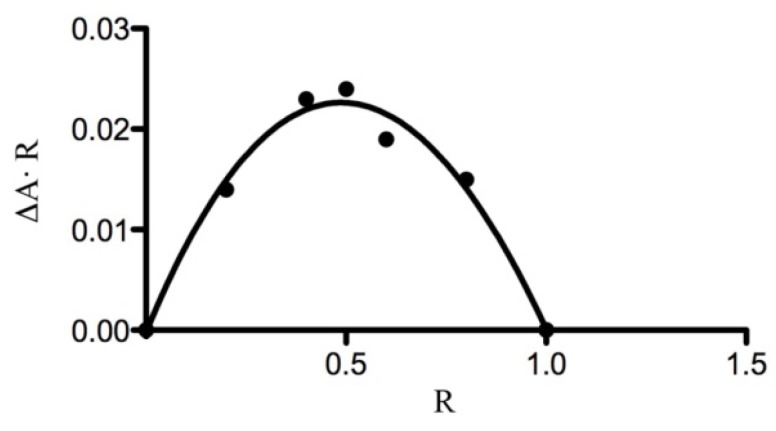
Job plot for the complex FeMeOH:β-CD (λ = 436 nm).

The 1:1 stoichiometry of the complex is confirmed by the continuous variation method [33]. Figure 4 presents the Job plot for the complex formed between FeMeOH and β-CD. In this curve, the position of the maximum is at R = [β-CD]/([FeMeOH] + [β-CD]) = 0.5, corresponding to a complex with 1:1 stoichiometry.

**Figure 5:**
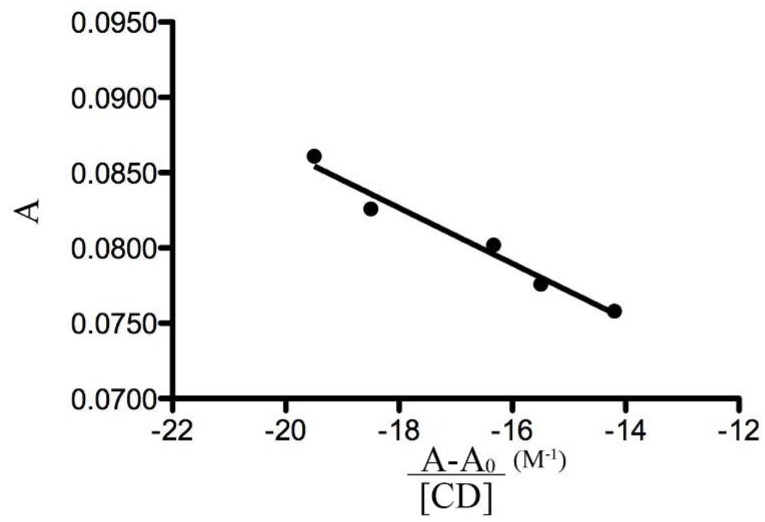
Dependence of FeMeOH absorbance from β-CD concentration (λ = 436 nm).

The evaluation of stability constants by direct spectroscopic methods relies on analytical differences between the free and complexed guest [34]. Changes in the absorption intensity of FeMeOH at 436 nm were monitored as a function of β-CD concentration to measure the binding constant. To conveniently calculate the stability constant (K_b_) we needed to rearrange the Benesi-Hildebrand equation [35] into a straight line form:

(1) $$A=-\frac{1}{K_{b}}\;\frac{A-A_{0}}{\left[\beta -\mathrm{CD}\right]}+A_{0}+\Delta \varepsilon \left[G\right]$$

where A and A_0_ are the absorbance of FeMeOH in the presence and absence of β-CD, respectively, K_b_ is the stability constant, [β-CD] and [G] are the concentrations of β-CD and FeMeOH, respectively and Δε is the difference in the molar absorptivities between free and complexed FeMeOH. Therefore, a plot of A *versus* (A − A_0_)/[β-CD] (Figure 5), gives a straight line with slope −1/K_b_. The K_b_ calculated value is 541.3 M^−1^.

#### 2.3.1. Phase Solubility Studies

**Figure 6:**
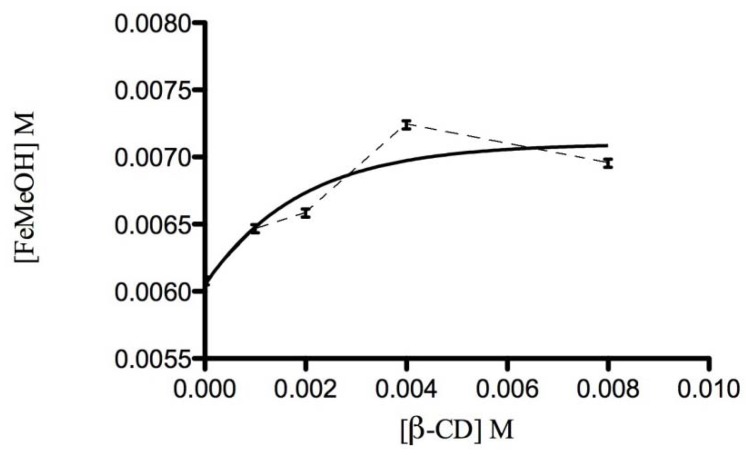
PSD for the complex FeMeOH:β-CD.

Solubility studies detect complex formation in solution taking into account the change of solubility of the guest substance as a function of the host concentration. The stoichiometric ratios and stability constant of the FeMeOH:β-CD were obtained by measuring the changes in UV-vis absorbance of the FeMeOH in the presence of increasing concentrations of the β-CD. The PSD for the complex formation between FeMeOH and β-CD is shown in Figure 6 and can be classified as Bs type according to Higuchi and Connors [36]. The FeMeOH solubility is enhanced by the presence of the host; in particular, a linear increase of solubility for FeMeOH was observed up to 4 × 10^−3^ M of β-CD. The ascending portion of the Bs type curve indicates that the stoichiometry of the complex is 1:1; then a short plateau region indicates the formation of an insoluble or with different stoichiometry complex in the solution at high concentrations of β-CD.

Rigorous nonlinear regression of experimental data was conducted [37,38] to obtain estimates of apparent stability constant (K_b_). Data analysis and nonlinear regression curve fitting were performed using Prism 5 software (GraphPad, San Diego, CA, USA). The value of K_b_ was found to be 528.0 M^−1^. A stability constant between the range of 100 and 1,000 M^−1^ is considered an ideal value, as smaller values indicated weak interactions between guest and CD; while a large value indicates incomplete guest release from the inclusion complex [39]. If the complex is too weak, there is little improvement in the solubility of the guest. On the other hand, if the complex is too strong, as indicated by a stability constant greater than 1,000 M^−1^, the complex cannot dissociate easily. In this frame, the stability constant obtained for FeMeOH:β-CD indicates that this complexation improves the bioavailability of FeMeOH [40]. 

### 2.4. Nuclear Magnetic Resonance (NMR)

Exploiting the changes in chemical shifts and in the hydrodynamic properties caused by the guest and the host on each other, the formation of FeMeOH:β-CD inclusion complex has also been investigated via NMR. 

1D ^1^H, 2D ^1^H-^1^H NOESY and 2D ^1^H-^1^H ROESY experiments were carried out on the CP sample in which the molar ratio between host and guest is 1:1. H3’ and H5’ of β-CD point toward the interior of the cavity with H3’ located near the wider edge and H5′ near the narrower edge that bears also the methylene (H6’) group. Here, only H3’ signals (3.85 ppm in the free β-CD spectrum, triplet) in β-CD show significant down-field shifts (3.90 ppm) upon interaction with FeMeOH. H5’ resonances are less affected by this interaction indicating that the host is not deeply inserted in the cyclodextrin cavity. The H6’ atoms and all the other hydrogens belonging to the CD are located on the exterior of the cavity and are therefore affected to a lesser extent by the presence of the guest. Moreover, a cross peak between the FeMeOH signal (4.2 ppm) and H3’ of β-CD indicate that the FeMeOH may be less than 5Å apart from the H3’ cyclodextrin hydrogens.

A series of Diffusion Ordered SpectroscopY (DOSY) experiments have been also carried out at various β-CD and FeMeOH molar ratio. In presence of an excess of FeMeOH, two clear populations of signals are visible that give (4.5 ± 0.2)·10^−10^ m^2^s^−1^ (FeMeOH signals) and (2.4 ± 0.2)·10^−10^ m^2^s^−1^ (FeMeOH:β-CD signals) translational diffusion coefficients, D_trans_, respectively. When the two compounds are in a 1:1 ratio, the DOSY experiments give only one set of signals with an average D_trans_ of (2.6 ± 0.2)·10^−10^ m^2^s^−1^. This behaviour further confirms the 1:1 stoichiometry of the FeMeOH:β-CD inclusion complex.

**Figure 7:**
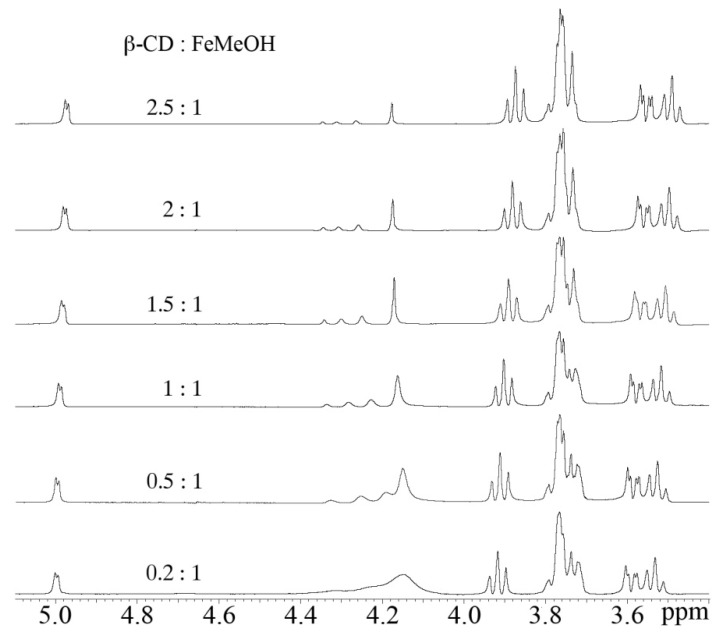
The ^1^H NMR spectra of β-CD and FeMeOH in different molar ratio.

Because the signal of the H3’ proton is well resolved and considerably intense, the differences of its resonance frequencies at various β-CD and FeMeOH molar ratio (Figure 7) were also exploited to calculate the K_b_ of the inclusion process. The chemical shift changes of H3’ *versus* the molar ratio of FeMeOH gave a good fit with a model involving a 1:1 complex [41,42] and the K_b_ calculated from these data yields a value of 479 M^−1^.

### 2.5. Cytotoxicity Test

**Figure 8:**
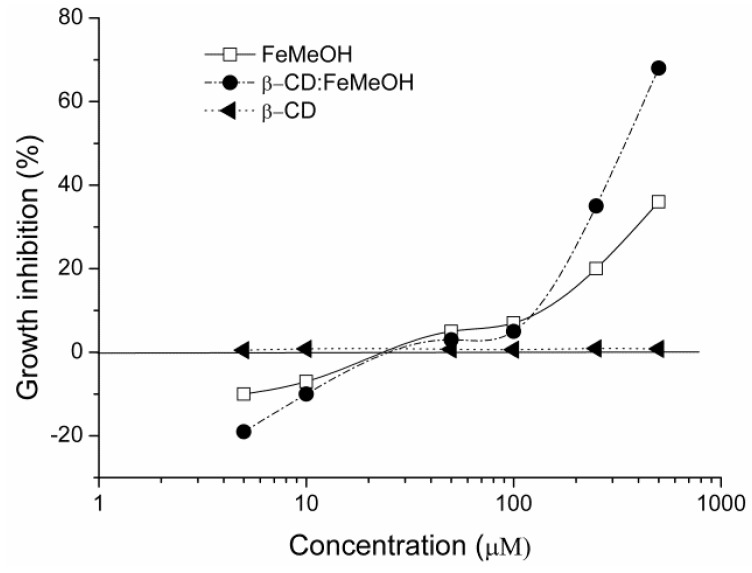
Growth inhibition of the compounds investigated on MDA-MB-231 cell line.

The results of the cytotoxicity tests are shown in Figure 8. Three independent experiments were performed with concentrations ranging from 5 to 500 μM. Compound concentrations used in the definitive tests were based on the results from range-finding tests. The MTT assay is based on the total mitochondrial activity which is related to the number of viable cells. Then, an increase or decrease of viable cells is linearly related to the mithocondrial activity that determines the conversion of the tetrazolium salt MTT into formazan crystals. Both FeMeOH and CP did not show any inhibition of the cell growth up to the concentration of 50 μM but at 250 and 500 μM, the CP determined a sharp increase of the ability to inhibit the cancer cell growth when compared to FeMeOH itself. In our experiments, the FeMeOH reached a growth inhibition of 36% at the maximum concentration tested (IC_50_ > 500 μM) through 48 h continuous treatments of cells with different concentrations of the compound and our results confirm those of Shago *et al.* [24] who reported an IC_50_ > 100 μM against HeLa cancer cells but after seven days of incubation. No difference in the growing capability was detected with β-CD when tested alone. Only for the inclusion complex it was possible to determine the IC_50_, the concentration able to inhibit the 50% of the cell population and the value was 344.7 (299.5–406.9) μM with a good dose-activity relationship in the range 100–500 μM. Our results agree with reported by Osella and co-workers [43] who used several ferrocene derivatives on Ehrlich ascites tumor cells and showed that the inclusion complex with β-CD increased the transport of ferrocene through the cellular membranes improving its cytotoxic activity. So we could assume that the inclusion of FeMeOH in β-CD confers the same important advantage.

## 3. Experimental 

### 3.1. Materials

FeMeOH and β-CD were purchased from Sigma-Aldrich. All the reagents and solvents were of analytical grade. Double distilled and MilliQ water was used throughout the experiments. 

### 3.2. Preparation of Solid Binary Systems 

The preparation of FeMeOH:β-CD solid inclusion complex was performed in 1:1 molar ratio by different methods, which are described below in detail. 

#### 3.2.1. Physical Mixing 

The PM was prepared by mixing powders of β-CD (0.262 g) and FeMeOH (0.05 g) in a mortar, at room temperature for 5 min. 

#### 3.2.2. Kneading Method

KND was obtained by adding a small volume of a water–methanol (50/50, v/v) solution to the FeMeOH (0.04 g) and β-CD (0.2 g) physical mixture and kneading the resultant mixture thoroughly with a pestle to obtain a homogeneous paste and continuing until the solvent was completely removed. The sample was dried at 40 °C in an oven for 30 min to remove traces of solvent. The dried mass was pulverized.

#### 3.2.3. Coprecipitation Method 

FeMeOH (0.02 g) was dissolved in methanol (250 µL) and added while stirring to a solution of β-CD (0.1 g) in water (25 mL) previously warmed to 60 °C. Stirring was maintained for 2 h at 60 °C. The system was gradually cooled to room temperature while stirring. After one night, the solution obtained was centrifuged at 4,000 rpm for 10 min. The precipitate was then placed in an oven at 60 °C for 2 h.

#### 3.2.4. Freeze-Drying Method

The required 1:1 stoichiometric quantity of FeMeOH (0.04 g) and β-CD (0.2 g) was mixing in a mortar for 15 min and then was dissolved in a water-methanol (50/50, v/v) solution (15 mL), while mixing with a magnetic stirrer. After methanol was removed, the residue was frozen at −40 °C for 24 h and then lyophilized in a freeze-dryer (Heto Drywinner 3-55) for 72 h. 

### 3.3. X-ray Powder Diffraction (XRD)

XRD patterns of FeMeOH, β-CD, binary systems and PM were obtained at room temperature using a Bruker AXS D8 Advance diffractometer (Karlsruhe, Germany) with tube anode Cu and a graphite monochromator using a voltage of 40 kV and a current of 30 mA. The diffractograms were recorded in the 2θ angle range between 3 and 30° and process parameters with scanning speed 0.05 θ/s. 

### 3.4. Fourier Transform Infrared Spectroscopy (FT-IR)

FT-IR analysis was performed on Perkin Elmer Spectrum GX spectrometer (Waltham, MA, USA). FT-IR measurements of the pure materials (FeMeOH and β-CD), binary systems and PM were carried out using KBr disks method. The KBr disks were prepared by compressing the powder. The scanning range was kept from 4,000 to 400 cm^−1^, resolution was 1 cm^−1^.

### 3.5. Ultraviolet-Visible (UV-Vis) Spectroscopy

For all UV-Vis spectroscopy studies, a UV-1700 Spectrometer (Shimadzu, Tokyo, Japan) was used with 1 cm matched quartz cuvettes. All measurements were recorded in the wavelength range 200–500 nm at room temperature.

The stoichiometry of the complex was determined using the continuous variation method [33]. According to this method, 1 mM solutions of FeMeOH and β-CD were mixed at different concentration ratios R = [β-CD]/([FeMeOH] + [β-CD]) keeping the volume constant. The stoichiometric ratio was obtained by plotting ΔA⋅R against R (where ΔA is the difference of absorbance of FeMeOH without and with β-CD) and finding the R value corresponding to the extreme of this dependence. 

To calculate the stability constant, the FeMeOH concentration was kept constant (1 mM) and β-CD concentration was varied (0–1 mM) and absorbance of the resulting solutions were measured. To conveniently calculate K_b_, we need to rearrange the Benesi-Hildebrand into straight line form, obtaining the Equation 1.

#### 3.5.1. Phase Solubility Study

Phase solubility studies were performed according to the method reported by Higuchi and Connors [36]. FeMeOH, in amount (30 mg) that exceeded its solubility, was taken into vial in which unbuffered MilliQ water (5 mL) containing various concentrations of β-CD (1–8 mM). These flasks were sealed and thermostatically shaken at 40 °C for 72 h. This amount of time is considered sufficient to reach equilibrium. Subsequently, the aliquots were withdrawn, using a syringe and samples were filtered immediately through a 0.45 µm Millipore membrane filter. A portion of the sample was analyzed by UV spectrophotometer at *λ*_max_ 436 nm, wavelength at which it was measured the FeMeOH specific molar absorbance after construction of the calibration curve. The solubility experiments were performed in triplicate. The total concentration of FeMeOH solubilized was calculated as: [FeMeOH] = A_FeMeOH_/ε_FeMeOH_ where A_FeMeOH_ is the phase solubility test absorbance and ε_Fe__Me__OH_ is the specific molar absorbance of FeMeOH at 436 nm. It is implicitly assumed in the Higuchi and Connors [36] procedure that the ε_FeMeOH_ value does not change upon complexation with β-CD [44]. Analysis of the PSD to obtain estimate of the complex formation constant, corresponding to the measured Bs type PSD, was carried out utilizing rigorous nonlinear regression curve fitting [37] which was performed using Prism 5 software (GraphPad, San Diego, CA, USA).

### 3.6. Nuclear Magnetic Resonance (NMR)

NMR spectra of cyclodextrin were recorded at 500 MHz using a Varian UNITY 500 spectrometer (Palo Alto, CA, USA). Deuterated D_2_O (99.9% relative isotopic abundance) was purchased from Cambridge Isotope Laboratories. In the titration experiment different aliquots of a 16 mM solution of βCD in D_2_O were progressively added to a 1 mM solution of FeMeOH in D_2_O. The proton chemical shifts were collected at 298 K and at pH = 5.6 and referenced to external TMS (δ = 0 ppm). Two-dimensional phase-sensitive NOESY, ROESY spectra [45] were collected using the States and Haberkorn method. All the experiments were typically acquired with a spectral width of 6,000 Hz, using 32–64 scans, 2D experiments were acquired with 256 increments and 2,048 data points in t_2_. Squared-shifted sine-bell functions were applied in both dimensions before Fourier transformation and baseline correction. NOESY and ROESY experiments were recorded with mixing times of 200 and 150 ms, respectively. Water suppression, when necessary, was achieved utilizing the DPFGSE sequence [46]. The data were processed and analyzed using the VNMRJ and CARA software [47]. The translation diffusion coefficient (D_trans_) was measured by using the pulsed-field gradient spin-echo DOSY (Diffusion Ordered SpectroscopY) experiments [48].

### 3.7. Cytotoxicity Test

The cell viability test (MTT assay) was carried out according to Zhang and coworkers [49]. Human breast cancer cell line MDA MB-231 was kindly provided by Prof. Ciro Abbondanza, Dipartimento di Patologia Generale, Seconda Università degli Studi di Napoli, Italy. For routine maintenance the cells were grown in Roswell Park Memorial Institute (RPMI) supplemented with 10% fetal bovine serum (FBS), 1% penicillin/streptomycin (10,000 U/mL), 2% L-Glutamine, 2% HEPES (Lonza, Verviers, Belgium), at 37 °C in an atmosphere of 5% CO2/95% air under saturating humidity. Cells were allowed to grow to confluence over 72 h starting of the tests. Each of the six doses (5, 10, 50, 100, 250, 500 μM) of FeMeOH and its complexation product was tested in four replicates and a negative control was included in each plate. In addition, the test was performed on the β-CD alone at the same concentrations. After 48 h of incubation, the cell growth inhibition was measured using the 3-(4,5-dimethylthiazol-2-yl)-2,5-diphenyltetrazolium bromide (MTT) test, based on the cleavage of the yellow tetrazolium salt to purple formazan crystals and after 4 h of incubation, spectrophotometrically quantified at 590 nm (Spectrafluor, Tecan, Parma, Italy). Cell viability rate was calculated as compound absorbance − control absorbance/control absorbance × 100. 

## 4. Conclusions 

The inclusion complex of FeMeOH with β-CD was prepared in the solid state by different techniques; the formation of complex was confirmed by XRD and FT-IR. The 1:1 inclusion complex formation in aqueous solution was determined by Job plot method and confirmed by NMR. The K_b_ of the complex was determined via NMR titration and it yields a value of 478 M^−1^ which is in agreement with that obtained by UV-Vis tritation (K_b_ = 541.3 M^−1^). Furthermore, utilizing PSD data, the stability constant was estimated by a rigorous nonlinear regression analysis and was found equal to 528.0 M^−1^. The PSD was classified as B_S_ type and it indicated that the solubility of FeMeOH was slightly increased in the presence of β-CD. The cytotoxic activity of CP showed that the inclusion complex has a higher capability in inhibiting cell growth if compared to that of FeMeOH free, because the inclusion allows a better transport of the FeMeOH to the cellular membrane. As a consequence, the benefits are not only the increase of the aqueous solubility but also the enhancement of FeMeOH cytotoxic activity, closely related to it, upon the complexation with β-CD. These results may be used to develop new compounds involved in analytical, catalytic, bioorganometallic or medical applications.
